# Development of Novel Surface-Enhanced Raman Spectroscopy-Based Biosensors by Controlling the Roughness of Gold/Alumina Platforms for Highly Sensitive Detection of Pyocyanin Secreted from *Pseudomonas aeruginosa*

**DOI:** 10.3390/bios14080399

**Published:** 2024-08-19

**Authors:** Waleed A. El-Said, Tamer S. Saleh, Abdullah Saad Al-Bogami, Mohmmad Younus Wani, Jeong-woo Choi

**Affiliations:** 1 Department of Chemistry, College of Science, University of Jeddah, P.O. Box 80327, Jeddah 21589, Saudi Arabia; 2 Department of Chemical and Biomolecular Engineering, Sogang University, 35 Baekbeom-Ro, Mapo-Gu, Seoul 121-742, Republic of Korea; jwchoi@sogang.ac.kr

**Keywords:** surface-enhanced Raman spectroscopy, biosensors, roughness, pyocyanin, *Pseudomonas aeruginosa*, gold/alumina platforms

## Abstract

Pyocyanin is considered a maker of *Pseudomonas aeruginosa* (*P. aeruginosa*) infection. Pyocyanin is among the toxins released by the *P. aeruginosa* bacteria. Therefore, the development of a direct detection of PYO is crucial due to its importance. Among the different optical techniques, the Raman technique showed unique advantages because of its fingerprint data, no sample preparation, and high sensitivity besides its ease of use. Noble metal nanostructures were used to improve the Raman response based on the surface-enhanced Raman scattering (SERS) technique. Anodic metal oxide attracts much interest due to its unique morphology and applications. The porous metal structure provides a large surface area that could be used as a hard template for periodic nanostructure array fabrication. Porous shapes and sizes could be controlled by controlling the anodization parameters, including the anodization voltage, current, temperature, and time, besides the metal purity and the electrolyte type/concentration. The anodization of aluminum foil results in anodic aluminum oxide (AAO) formation with different roughness. Here, we will use the roughness as hotspot centers to enhance the Raman signals. Firstly, a thin film of gold was deposited to develop gold/alumina (Au/AAO) platforms and then applied as SERS-active surfaces. The morphology and roughness of the developed substrates were investigated using scanning electron microscopy (SEM) and atomic force microscopy (AFM) techniques. The Au/AAO substrates were used for monitoring pyocyanin secreted from *Pseudomonas aeruginosa* microorganisms based on the SERS technique. The results showed that the roughness degree affects the enhancement efficiency of this sensor. The high enhancement was obtained in the case of depositing a 30 nm layer of gold onto the second anodized substrates. The developed sensor showed high sensitivity toward pyocyanin with a limit of detection of 96 nM with a linear response over a dynamic range from 1 µM to 9 µM.

## 1. Introduction

Recently, metal nanoparticles (NPs) have been promising agents for a wide range of applications, including biosensors and drug delivery systems. Due to the unique capability of metal NPs to interact with biomolecules, various NPs were used for early disease diagnosis and treatment. *Pseudomonas aeruginosa* (*P. aeruginosa*) bacteria could produce infections in patients with high mortality rates. *P. aeruginosa* infection is drug resistant, which could cause impaired vision, light sensitivity, and discharge from the eye [1,2,3,4,5]. Thus, early and accurate *P. aeruginosa* pathogen detection is the main reason for a more targeted antibiotic prescription [6,7]. Several techniques were reported for *P. aeruginosa* infection diagnosis; however, most are time consuming, costly, and need complicated sample preparations [8]. Several sensors were recently reported as efficient tools for *P. aeruginosa* identification based on monitoring the released pyocyanin (PYO) marker [8,9,10,11,12,13,14,15,16,17,18,19,20,21]. Therefore, increasing research is conducted to obtain relatively robust, accurate, and inexpensive methods, leading to several nanotechnology-based methods coupling electrochemical and optical readouts. The electrochemical and optical techniques are among the most widely applied due to their unique features [22]. Electrochemical sensors have unique advantages, high sensitivity, high selectivity, cheap, rapid, and direct measurement [6,7,8,9,10]. Although electrochemical approaches showed high sensitivity and selectivity, there is a need for developing a highly selective, specific, sensing method that could apply to a wide range of analyses.

The Raman technique showed unique advantages among the different optical techniques due to its fingerprint data, no sample preparation, and high sensitivity besides its ease of use. Surface-enhanced Raman scattering (SERS) has exhibited tremendous efficacy toward single molecule detection. The SERS enhancement factor critically depends on different parameters, including (i) the nature of the metal, (ii) the metal size, (iii) the shape of the metal, and (iv) the degree of the surface roughening [23]. Thus, the substrate preparation plays a central concern [24].

Using noble metal nanostructures to improve the Raman response is one strategy of SERS. However, the improvement is disadvantageous because it is not uniform in the impact of the nanoparticles on the biological samples. Uses of decorated substrates with metallic nanostructures as SERS-active surfaces is another strategy that has consistently demonstrated potential for qualitative and quantitative analyses of the species [8]. However, the formation of a uniformly modified surface is challenging since a complicated and expensive instrument is needed, such as a laser lithography machine. Recently, the fabrication of modified substrates based on 3D printers has been a potential route. However, the 3D printer is not available everywhere. Furthermore, the use of the 3D printer needs trained skills. Therefore, there is a need to develop a cheap, easy route for the fabrication of nanostructured modified substrates.

A rough surface could be generated based on different methods such as (i) electrochemical oxidation-reduction, (ii) chemical etching of the surface in acids, (iii) deposition of a colloid solution on the surface, or (iv) by depositing a metal film onto substrates either by electrochemical deposition, thermal evaporation, lithography, or sputtering [25]. All the above techniques lead to decorating the metal surface with tiny metal particles, which act as metal roughness. The size and shape of the roughness features will affect the laser wavelength as well. The surface roughness features (tens of nanometers) are usually much smaller than the wavelength of the excitation radiation (i.e., the incident laser), which allows the excitation of the surface plasmon to be localized on the surface and hence results in an electromagnetic field enhancement.

Thus, much attention has been given to studying and controlling the surface roughness. Most methods produce heterogeneous surfaces with nonuniform sizes, shapes, and orientations [26]. A controllable and reproducible metal particle deposition was developed to control the roughness features [26,27].

Nanoporous anodic aluminum oxide (AAO) substrates are among the best hard templates because of their unique optical/electrochemical features as well as their ability to produce vertically aligned and highly uniform nanoporous structures based on an easy, simple, and cost-effective electrochemical fabrication process [28]. Thus, AAO has a wide range of applications, such as template synthesis, molecular separation, catalysis, energy production/storage, electronics, photonics, biosensing, and drug delivery [29,30].

Recently, AAO substrates have been used for developing SERS-active substrates based on the evaporation of silver or gold NPs onto the AAO substrate. The size and the inter-distance of the fabricated nanostructures could be controlled based on the AAO pore geometry and the metal deposition conditions, hence enabling the specific sensing applications of these SERS-AAO sensors.

**Scheme 1:**
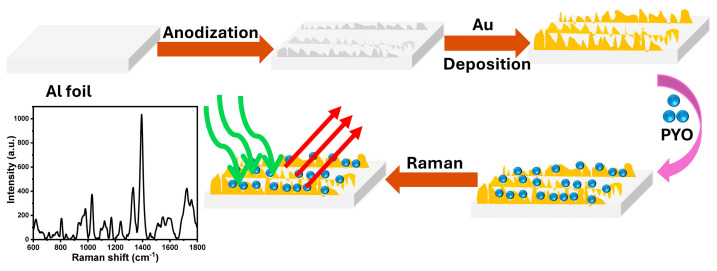
Design and fabrication of Au/AAO SERS-active surface for sensing PYO biomarker.

Here, we reported fabricating different AAO substrates with different roughness factor values. These substrates were coated with a thin layer of gold to create SERS-active surfaces and a biocompatible surface for immobilizing different targeting biomolecules as biosensing devices. The effect of gold layer thickness was investigated. Morphologies of the developed AAO and Au-coated AAO substrates were investigated based on scanning electron microscope (SEM) and atomic force microscopy (AFM) techniques. Moreover, AFM was used to study the roughness of the different AAO substrates before and after coating with a layer of Au of different thicknesses. The modified surfaces were applied as a SERS-based biosensor for detecting low concentrations of PYO biomarkers in different samples (Scheme 1).

## 2. Materials and Methods

### 2.1. Chemicals

Aluminum foil (99.99%, 100 µm thickness, Tokai, Osaka, Japan), HClO_4_, ethanol, oxalic acid, phosphoric acid, chromic acid, pyocyanin (P0046-5MG), and mercaptobenzoic acid (MBA) were purchased from Sigma (St. Louis, MO, USA). Lysogeny broth (LB) was obtained from BIO BASIC INC, Markham, Canada.

### 2.2. Apparatus

Field emission scanning electron microscopy [FESEM; Hitachi S-4700, Tokyo, Japan] was used to study the morphology of different AAO and AAO/Au-coated substrates. The morphology and roughness of the fabricated substrates were investigated by AFM (NTEGRA spectra, AFM-Raman Spectrometer, NT-MDT, Moscow, Russia). A semi-contact mode was performed using an NSG01 cantilever with a force constant of 2.5 to 10 N/m, a resonant frequency within a range of 115–190 kHz, and a scan rate of 1 Hz. In addition, the Raman spectra were recorded using the NIR laser emitting at 785 nm with an irradiation laser power of 3 mW on the sample plane. Ten scans of 10 s were recorded within the Raman shift range from 600–1800 cm^−1^, and the mean data were used.

### 2.3. Fabrication of SERS-Active Surfaces

To control the substrates’ roughness, different degrees of anodization were fabricated. The substrates were fabricated as follows: (1) electropolishing the aluminum foil using a mixture of HClO_4_ and ethanol (1:5, *v*/*v*) under a constant voltage of 20 V for a minute. (2) Then, the first anodization step was carried out in oxalic acid (0.3 M) under a DC voltage of 40 V at 3 °C for 8 h. (3) The alumina layer that formed during the previous step was dissolved in a mixture of phosphoric acid (0.4 M) and chromic acid (0.2 M) at 65 °C for 6 h. (4) Then, the second anodization process was conducted under the same conditions as for the first anodization but for only 4 min. (5) The formed alumina layer was slightly etched by immersing the substrate in phosphoric acid (5 wt.%) for 12 min at 30 °C. (6) Layers of Au with different thicknesses (20 nm, 30 nm, and 50 nm) were deposited onto the AAO substrates based on a thermal evaporator (ULVACVPC-260) with an evaporation rate of about 0.1 Å/s under a vacuum pressure of 3 × 10^−6^ Torr [29].

### 2.4. Investigate the Substrates’ Roughness

The roughness of the fabricated substrates was measured using the AFM technique. The AFM scan was performed for each substrate at a scan rate of 1 Hz over a scan area of 1 µm^2^. Nova (1.0.26.1484) software was used to analyze the roughness. The histogram of each AFM scan was obtained, and then all the roughness parameters were obtained.

### 2.5. Pseudomonas aeruginosa Clinical Isolates

*P. aeruginosa* cultures were made from clinical isolates obtained from the Medical Microbiology and Immunology Department at Assiut University. *P. aeruginosa* cultures were made according to the reported protocol [9,10]. The study protocol was approved by the local Ethical Committee of the Faculty of Medicine, Assiut University (IRB no: 17300293), and informed written consent was taken from all the study participants.

### 2.6. PYO Sensing in Pseudomonas aeruginosa Cultures Based on SERS Technique

A colony of *P. aeruginosa* was added to a 14 mL tube containing 10 mL LB under sterile conditions. Then, the tube was placed on a shaker (200 rpm) at 37 °C overnight, and 1 mL of this suspension was transferred to 9 mL of fresh LB broth and kept in a shaker for a further day at 37 °C. The OD at 600 nm (OD600) was measured to quantify the density of the bacteria and confirm the increasing bacterial number in each culture sample. The PYO was collected after 24 h and investigated using different modified substrates based on the SERS technique. A 50 µL sample of the collected PYO from each culture sample was immobilized on the surface of each modified substrate for 6 h. The substrates were rinsed with deionized water to remove non-immobilized PYO molecules and dried using N_2_ gas.

## 3. Results and Discussion

### 3.1. The Rough Substrate Fabrication

**Figure 1:**
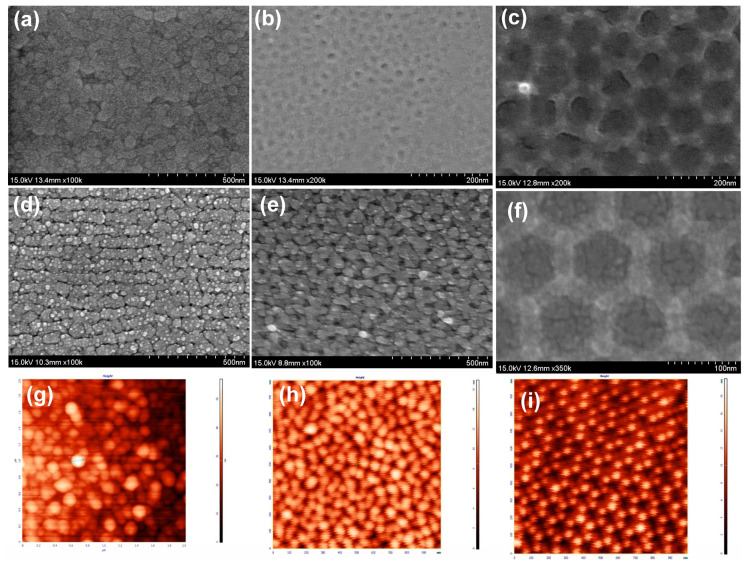
(**a**) SEM image of the electro-polished aluminum substrate, (**b**) SEM image of aluminum foil after first anodization, (**c**) SEM image of aluminum foil after second anodization, (**d**) SEM image of Au/electro-polished aluminum substrate, (**e**) SEM image of Au/first anodized aluminum foil, (**f**) SEM image of Au/second anodized aluminum foil, (**g**) AFM image of Au/electro-polished aluminum substrate, (**h**) AFM image of Au/first anodized aluminum foil, and (**i**) AFM image of Au/second anodized aluminum foil.

Aluminum foil was used for fabricating several substrates with different roughness degrees based on the anodization process [5]. The degree of the anodization was used to control the degree of the roughness as follows. (1) The first substrate was prepared by electropolishing the aluminum foil for 60 s under a constant voltage (20 V) in a solution of HClO_4_ and ethanol (1:5, *v*/*v*). The electropolished substrate’s morphology was investigated by using the SEM technique. Figure 1a shows the SEM image that indicated the presence of Al nanoparticles with a diameter of about 70 nm. (2) The first anodization process was achieved in a 0.3 M oxalic acid solution under a DC voltage of 40 V for 8 h at 3 °C; followed by dissolving the formed alumina layer during the first anodization process using a mixture of phosphoric acid (0.4 M) and chromic acid (0.2 M) for 6 h at 65 °C. The morphology of the Al substrate after first anodization was studied using SEM (Figure 1b), which indicated the formation of porous structures with a pore diameter of 20 nm. (3) Then, the second anodization process was conducted for 4 min under the same conditions as that for the first anodization. After completing the second anodization, the alumina layer was etched slightly by immersing the substrates in an aqueous solution of phosphoric acid (5 wt. %) at 30 °C for 12 min. The SEM figure after the second anodization process showed the formation of porous nanostructures with an average pore diameter of about 40 nm (Figure 1c). (4) Finally, a layer of Au NPs (20 nm) was deposited onto the surface of the fabricated substrates (electropolished, first anodized, and second anodized Al substrates) based on the thermal evaporation method [29]. The thickness of the Au layer was controlled by the evaporation time. The thermal deposition of a 20 nm layer of Au onto the electropolished, first anodized, and second anodized Al substrates showed the formation of tiny Au NPs as shown in the SEM and AFM images (Figure 1d–i).

**Figure 2:**
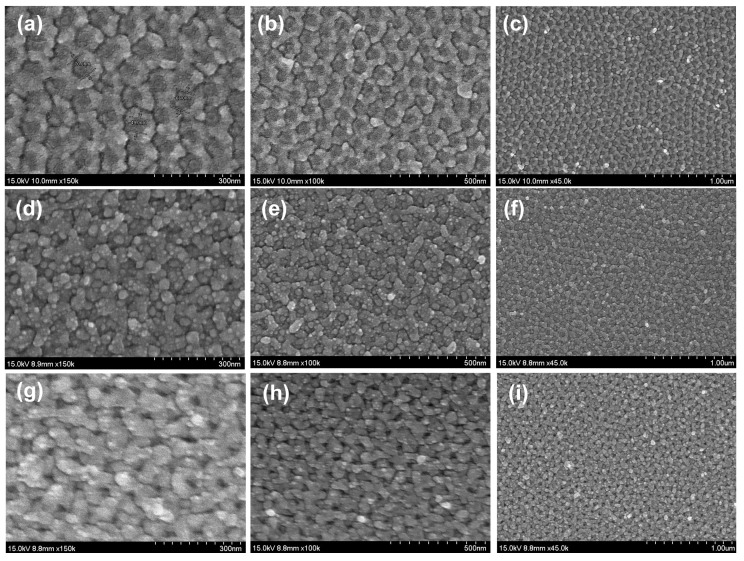
SEM image with different magnifications of second anodized aluminum foil after depositing (**a**–**c**) 20 nm of Au, (**d**–**f**) 30 nm of Au, and (**g**–**i**) 50 nm of Au.

To investigate the effect of high Au on the Raman effect and the surface roughness, the Au layer was deposited onto the second anodized Al substrates with three different thicknesses (20, 30, and 50 nm). Figure 2 shows different magnifications of the SEM images after the deposition of an Au layer onto the second anodized Al substrates. Figure 2a–c shows the SEM images of the second anodized Al substrate coated with a 20 nm layer of Au that showed the growth of a very tiny Au particle with an average size of 12.67 nm. Furthermore, the SEM images of the second anodized Al substrate coated with a 30 nm layer of Au were represented in Figure 2d–f, which indicated the presence of Au NPs with an average size of 19.53 nm. Moreover, Figure 2g–i showed the SEM images of the second anodized Al substrate coated with a layer of Au (50 nm in thickness). The data showed the formation of Au NPs with an average particle size of about 23.79 nm.

### 3.2. AFM Analysis of the Surface Roughness

**Figure 3:**
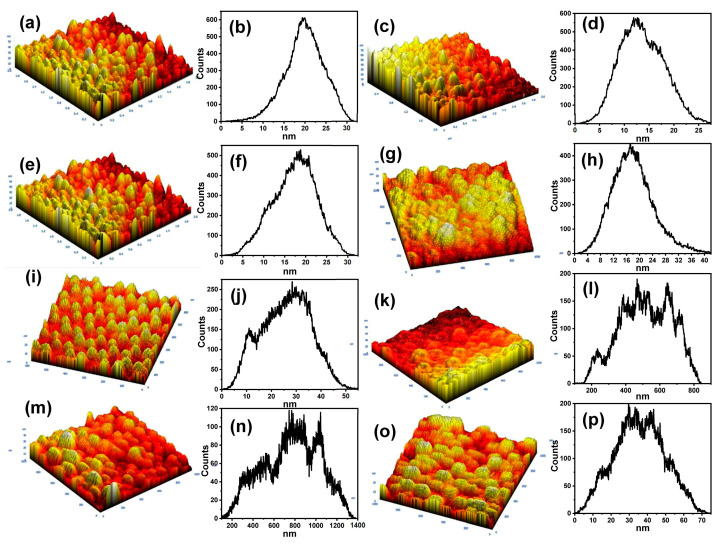
(**a**) 3D AFM of electro-polished Al foil, (**b**) histogram of electro-polished Al foil, (**c**) 3D AFM of Au_20nm_ modified electro-polished Al foil, (**d**) histogram of Au_20nm_ modified electro-polished Al foil, (**e**) 3D AFM of first anodized Al foil, (**f**) histogram of first anodized Al foil, (**g**) 3D AFM of Au_20nm_ modified first anodized Al foil, (**h**) histogram of Au_20nm_ modified first anodized Al foil, (**i**) 3D AFM of second anodized Al foil, (**j**) histogram of second anodized Al foil, (**k**) 3D AFM of Au_20nm_ modified second anodized Al foil, (**l**) histogram of Au_20nm_ modified second anodized Al foil, (**m**) 3D AFM of Au_30nm_ modified second anodized Al foil, (**n**) histogram of Au_30nm_ modified second anodized Al foil, (**o**) 3D AFM of Au_50nm_ modified second anodized Al foil, and (**p**) histogram of Au_50nm_ modified second anodized Al foil.

AFM is a powerful technique for studying the roughness properties and the morphology of the developed surfaces [29]. Here, a tapping mode was used to perform the AFM of the fabricated surfaces and to avoid scratching of the surface. The roughness of the Al foil after each step of the anodization was investigated before and after the deposition of the Au NPs. To calculate the roughness, an AFM image of each substrate was recorded at a scan rate of 1.0 Hz and the scan configuration of 512 × 512 pixels. Then, the roughness parameters were calculated from the corresponding histogram. Figure 3 shows the AFM images and the histograms of the developed substrates before and after being coated with Au layers of different thicknesses. The 3D topography of the electropolished substrate showed the presence of large particles (Figure 3a), which were reduced with the depositing of the Au layer, and the height was increased (Figure 3c). These results were confirmed by the roughness values as shown in Figure 3b,d, which confirmed the increase in the surface roughness after depositing the Au NPs. The AFM image of the 3D topography of the first anodized Al foil confirms the formation of hollow structures (Figure 3e). On the other hand, the 3D topography of the first anodized Al after depositing the Au layer indicates the reduction of the size of the pores (Figure 3g). Moreover, the 3D topography of the second anodized Al foil (Figure 3i) showed the formation of a honeycomb pattern. The morphologies of the different Al substrates after depositing the Au layer are shown in Figure 3k,m,p. The roughness histograms of the first and second anodized Al substrates before and after deposited Au layers are represented in Figure 3. The 3D AFM images showed a difference in colors that related to the difference of the heights. The roughness evaluation parameters of the fabricated substrates were obtained from the corresponding histograms and are shown in Table 1. The roughness evaluation parameter data of the developed substrates indicated that the roughness parameters depend on the type of anodization as well as the thickness of the Au layer.

### 3.3. Effect of the Surface Roughness on the Raman Enhancement of the Different Substrates

**Figure 4:**
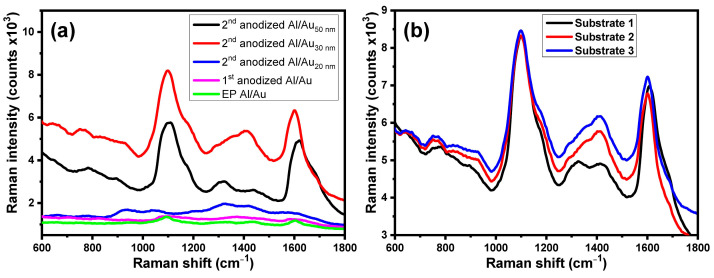
(**a**) SERS spectra of MBA immobilized on (green curve) Au_20nm_ modified electro-polished Al substrate, (pink curve) Au_20nm_ modified first anodized Al substrate, (blue curve) Au_20nm_ modified second anodized Al substrate, (red curve) Au_30nm_ modified second anodized Al substrate, and (black curve) Au_50nm_ modified second anodized Al substrate, and (**b**) SERS spectra of MBA immobilized on three Al/Au_30nm_ substrates.

The roughness degree effect on the Raman enhancement efficiency of the different developed substrates was investigated by studying the Raman signal of MBA that was immobilized on the modified substrates. To study the efficiency of the prepared substrates to enhance the Raman signals, 100 µL of MBA (1 mM) were immobilized over each substrate for 2 h and then the Raman signals were recorded. Figure 4a shows the Raman spectra of MBA immobilized at different substrates. The data demonstrated that the Raman signal intensity depends on the substrate type. The results indicated that among all tested substrates, the second anodized Al substrate coated with a 30 nm layer of Au has the highest Raman enhancement effect. It is worth noting that the surface of the second anodized Al substrate coated with a 30 nm layer of Au has the highest roughness and highest enhancement Raman effect. The rougher surface creates more hotspot centers, and these additional hotspots could add more Raman enhancement based on the electromagnetic mechanism. Furthermore, this surface will add more active spots for immobilizing the analyte molecules. Moreover, the rougher surface possesses a larger surface area that results in the formation of a monolayer of the analyte. Thus, this results in further Raman enhancement based on the chemical enhancement mechanism.

The reproducibility of the modified substrate was studied by fabricating three-second anodized Al/Au_30nm_ substrates and using them for sensing 1 mM of MBA. The Raman intensity shows little variation between the used substrates with a relative standard division (RSD) of about 2.43%, which indicates the high reproducibility of the fabrication process of the modified substrates.

### 3.4. SERS of PYO at the Designed Substrates

**Figure 5:**
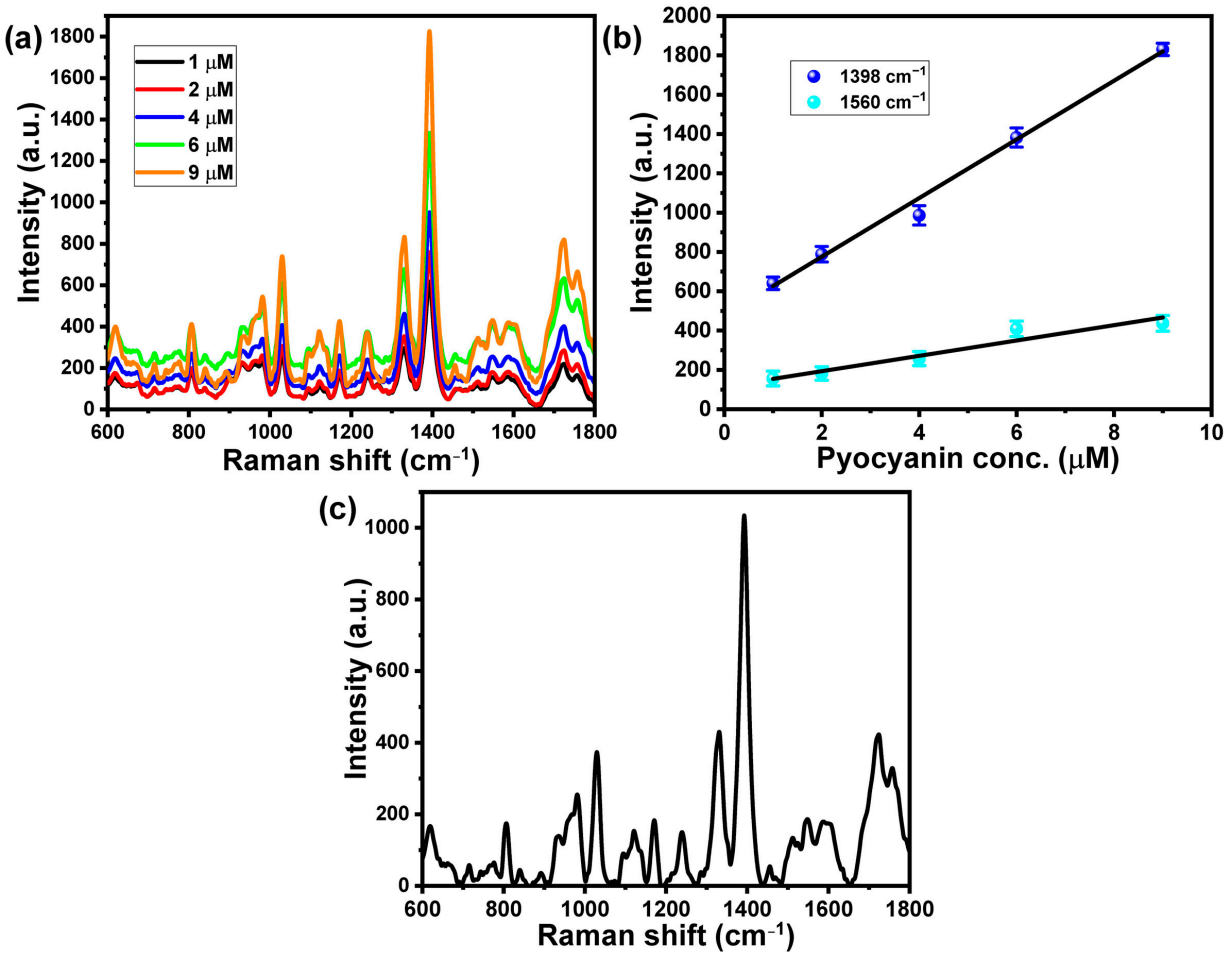
(**a**) SERS spectra of different concentrations of PYO using Au_30nm_ modified second anodized Al substrate, (**b**) relationship between the intensity of Raman peak at 1398 cm^−1^ and the PYO concentration using Au_30nm_ modified second anodized Al substrate, and (**c**) SERS spectrum of PYO released from *P. aeruginosa* sample using Au_30nm_ modified second anodized Al substrate.

Different PYO solutions were prepared with a concentration ranging from 1 µM to 9 µM, and each PYO solution was immobilized on the Al/Au_30 nm_ substrate for 2 hrs; then the substrate was washed and dried under N_2_ gas. Five SERS spectra were collected for each solution, and the mean data were used. Figure 5a shows the SERS spectra of the different solutions. The SERS spectra represented a set of characteristic peaks at 676, 1355, 1560, and 1598 cm^−1^ [30,31,32,33]. The peaks at 676, 1560, and 1598 cm^−1^ are attributed to the ring deformations [31]. Furthermore, the bands at 1355 and 1398 cm^−1^ are related to C-C stretching and in-plane C-H bending, also, a peak at 1355 cm^−1^ is assigned to the C-N stretching of the central aromatic ring [30]. The SERS technique was used for detecting a wide range of PYO concentrations from 1–9 µM, which showed increased intensities of the Raman peaks with increasing PYO concentrations. Figure 5b shows the relationship between the PYO concentrations and the intensity of the Raman peaks at 1398 and 1560 cm^−1^. The curves showed that the Raman peak intensities are linear depending on the PYO concentration over a dynamic range from 1 µM to 9 µM with an R^2^ of 0.9956 and 0.9321, respectively. Based on the R^2^ value, the calibration curve for the Raman peak at 1398 cm^−1^ was used to study the sensor sensitivity and to calculate the detection limit. The detection limit is 96 nM, lower than a tenth of the PYO concentration in clinical samples [19]. The sensitivity of the developed sensors was compared with the sensitivity of several electrochemical/spectroscopy-based sensors. The proposed sensor’s detection limit was compared with the previously reported data (Table 2). The results indicated that the LOD of the proposed sensor is lower than many of the previously reported sensors [9,19,34,35,36,37,38,39,40]. The high sensitivity of the current sensor, besides its ease of use and no need for sample preparation, are among the advantages of this sensor, which could open the door for real sample diagnosis and in-field uses. The Raman measurements were repeated 10 times, and the mean values were used. The repeatability of the proposed sensor was studied by calculating the standard deviation of the SERS results and the standard deviation of the mean. The repeatability of our method was found to be 0.000625, indicating the process’s high precision.

### 3.5. Sensing of PYO Secreted from Pseudomonas aeruginosa Based on SERS Spectroscopy

The second AAO/Au_30_ nm substrate was used as a SERS-active surface for detecting the PYO biomarker secreted from *Pseudomonas aeruginosa* bacteria. Figure 5c shows the Raman spectrum of PYO secreted from *Pseudomonas aeruginosa* bacteria, which represents the PYO characteristic peaks.

## 4. Conclusions

Here we established a new approach for developing SERS-based active substrates based on designed Au/AAO modified substrates. AAO substrates of different roughness were synthesized based on an anodization process. The application and performance of the different developed substrates serve as a platform for SERS-based biosensing devices. Furthermore, the obtained AAO substrates were decorated with Au NPs to enhance their Raman properties. The effect of the Au thickness was investigated. The obtained sensor was used for detecting a wide range of PYO in different samples. This sensor showed high sensitivity with an LOD of 0.96 µM. These substrates could be applied for multiplexed detection on a simple, easy-to-use, and highly sensitive SERS-based biosensor. Modification of AAO with Au NPs will open the door for a wide range of applications besides the role of the Au layer in enhancing the Raman effect.

## Figures and Tables

**Table 1:** The roughness parameters of the fabricated substrates.

	EP Al	EP Al/Au	1st Al	1st Al/Au	2nd Al	2nd Al/Au_20_	2nd Al/Au_30_	2nd Al/Au_50_
Sy (nm)	23.42	27.55	32.35	43.00	56.83	80.74	131.71	75.55
Sz (nm)	11.61	13.92	16.16	21.87	28.637	39.11	65.54	37.93
Average (nm)	11.14	13.54	17.31	17.98	25.88	31.22	58.43	35.67
Sa (nm)	3.01	3.61	4.16	5.01	8.15	12.02	21.26	11.13
Sq (nm)	3.66	4.43	5.15	6.40	9.86	14.38	25.74	13.59
Ssk	0.27	0.21	−0.20	0.53	−0.04	0.17	0.21	0.02
Ska	−0.48	−0.35	−0.34	0.44	−0.62	−0.76	−0.73	−0.54
Second moment	11.73	14.24	18.06	19.09	27.69	34.37	63.84	38.17
Entropy	7.25	7.55	7.77	8.07	8.69	9.18	10.03	9.16
Redundance	−0.61	−0.60	−0.56	−0.50	−0.50	−0.45	−0.43	−0.47

EP Al (electropolished Al foil), Sy (Peak-to-peak), Sz (Ten-point height), Sa (Average Roughness), Sq (Root Mean Square), Ssk (Surface skewness), and Ska (Coefficient of kurtosis).

**Table 2:** The sensitivity of the present sensor compared with previous research.

Technique	LOD (µM)	Reference
Cyclic voltammetry	0.5	[9]
Cyclic voltammetry	2	[34]
Square wave voltammetry	0.51	[35]
Square wave voltammetry	1	[35]
Square wave voltammetry	1.6	[35]
Square wave voltammetry	0.003	[36]
Square wave voltammetry	0.03	[37]
Square wave voltammetry	0.95	[38]
Differential plus voltammetry	0.05	[39]
Amperometry	0.125	[40]
Raman spectroscopy	0.5	[19]
Raman spectroscopy	0.096	Current study

## Data Availability

All relevant data are within the manuscript file.
